# Dietary Habits, Vitamin and Mineral Supplements in Patients with Chronic Kidney Disease (CKD)

**DOI:** 10.3390/nu12123817

**Published:** 2020-12-14

**Authors:** Piergiorgio Messa

**Affiliations:** 1 Unit of Nephrology, Università degli Studi di Milano, Via Commenda 15, 20122 Milano, Italy; piergiorgio.messa@unimi.it or piergiorgio.messa@policlinico.mi.it; Tel.: +39-0255034551 or +39-0255034552; Fax: +39-0255034550; 2 Nephrology, Dialysis and Renal Transplant Unit—Fondazione IRCCS Ca’ Granda Ospedale Maggiore Policlinico di Milano, Via Commenda 15, 20122 Milano, Italy

Chronic kidney disease (CKD) is frequently complicated with a malnutrition status, due to the presence of gastrointestinal symptoms and/or to dietary and multi pharmacological prescriptions which are almost universally present in such patients.

The altered nutritional status of CKD patients frequently includes changes of protein, lipid, and glucose metabolism, but it may also include an impaired availability of vitamins and other micronutrients.

Another emerging issue is related to the possibility that these complex nutritional modifications might be directly or indirectly related with the change in the quantitative and/or qualitative composition of the intestinal microbiota (MB), with a cause–effect relationship far from being well defined.

Most of these alterations have been considered to be directly or indirectly related to many relevant clinical complications which are commonly observed in CKD patients.

In this Special Issue of *Nutrients*, we collected a number of excellent contributions from experts who shared their experimental data and thoughtful opinions, built on their deep experience in the fields related to the above-mentioned nutritional issues.

I will limit myself to briefly report on a brief summary on each one paper collected in the present Issue, inviting you to go more in depth carefully reading these such highly informing articles.

Protein-energy wasting (PEW) is one of the most feared consequence of dietary protein restriction (DPR) which is a very frequent adopted prescription in the advanced stages of CKD, in an attempt to delay as longer as possible the start of dialysis treatment, improving uremic symptoms. To counteract the possible DPR-dependent PEW, some nephrologists add ketoanalogue (KA) supplementations to a low-protein diet (LPD). However, there is as yet limited evidence confirming the efficacy and, not less important, the safety of such an intervention, in particular in the long term. This issue was specifically explored by Yen CL and coworkers [1] who reported on the long term effects of a Low protein diet (LPD) supplemented with ketoanalogues, collecting data from a very large cohort of patients with advanced stages of CKD, included in the Taiwan National Health Insurance Research Database (NHIRD). The Authors report that CKD patients who followed a LPD supplemented with KA experienced a reduced risk for the all-cause mortality, for the mortality by major cardiovascular and cerebro-vascular causes, and for the infection related mortality, in comparison with patients not on supplemented LPD, encouraging in the use of such a dietary intervention.

Nephrologists are also well aware that both obesity and high lipid levels may adversely affect kidney function. Particular attention has been more recently payed to the potential nephrotoxic role of free fatty acid (FFA). Gai Z et al. [2], in an extensive and in-depth review of the literature, have crossed the topic starting from the hypothesized pathogenetic mechanisms up to the possible interventions aimed at correcting the toxic effect of FFA, passing from the correction of lifestyles to more specifically pharmacological interventions.

Increased consumption of energy-dense foods, characterized by a high caloric content, are considered among the most relevant causal factors of metabolic syndrome, obesity and hypertension. Since dietary counselling in CKD patients is often directed to maintain a high caloric intake, energy-dense foods could be sometime taken into account, introducing potential risk factors contributing to metabolic derangements which are commonly found in such patients. Mariano Rodriguez and Escolastico Aguilera-Tejero [3] faced the issue of the potential impact of energy-dense diets in particular on mineral metabolism in the context of CKD and mineral bone disorder syndrome. The main conclusions of the Authors, based on a critical evaluation of the most relevant papers published on this topic, are that there is a strong theoretical background for discouraging the use of an energy-dense diet in CKD patients, for its potential negative effects on most of the mineral metabolism parameters and on the bone health, on the inflammatory status, and also on the progression of CKD itself.

It has been known for a long time that the reduced renal synthesis of the most potent Vitamin D metabolite, calcitriol, plays an outmost role in the pathogenesis of secondary hyperparathyroidism (SHP) of CKD patients. In the last few decades, the role of the reduced levels of nutritional vitamin D (nVitD), cholecalciferol and ergocalciferol, has been more and more recognized as an additional potential pathogenic factor of SHP, also in this clinical setting. Lu CL et al. [4] performed a wide and accurate review of the published studies, addressing the role of nVitD deficiency in CKD patients. By this critical review of the literature, the Authors conclude that the correction of nVitD deficiency is effective in preventing and correcting SHP, particularly in the early stage of CKD.

Still in the field of interest relating to native vitamin D in patients with CKD, Alfieri C et al. [5] work with particular attention to the real or supposed roles of VitD defined as non-canonical roles, that is to say not strictly connected to mineral and bone metabolism. The Authors reinforce the awareness that it is worth correcting both native and active Vit D deficiency in CKD patients.

Following the progressive increase in aging in the general population, patients with CKD are also becoming older, showing signs of frailty with increasing frequency, with sarcopenia representing an overwhelming characteristic. This problem represents a critical issue in dietary managing of such patients, given the need of reducing protein intake, avoiding a reduction in the patient’s muscle mass. Claudia D’Alessandro and colleagues [6] carried out an observational study, submitting to a nutritional and functional assessment 40 CKD patients aged ≥75 years, compared with 40 CKD patients aged 60–74 years. The Authors found that sarcopenia was significantly more frequent in the older patients; however, the presence of sarcopenic conditions were associated with the degree of physical activity as well as age, but not with glomerular filtration rate levels or dietary intake. The Authors’ suggestion is that we can confidently use protein restriction in elderly CKD patients, but at the same time we should promote their physical activity.

In line with the topic addressed by the previous report, Fois A et al. [7] performed a feasibility study, proposing an individualized dietary approach, offering 131 patients with advanced stages of CKD the possibility of choosing among a normalization (0.8 g/kg b.w.) or a moderate reduction in their protein intake, this second choice performed with a “traditional” mixed protein diet or a “plant-based” protein diet plus ketoacids. Normalization of the diet was chosen by 57% of patients, while 18% chose the traditional protein regimen, 17% the plant-based protein diet, and the remaining 8% were excluded from the study for clinical reasons. The patients’ compliance to the dietary regimen, over an observation period longer than 3 months, was relatively high (74%), regardless of the chosen dietary regimen, supporting the feasibility of an individualized dietary approach.

On the other hand, it is not yet clear whether or not a protein restriction greater than 0.8/kg of b.w. has any impact on the mortality rate of CKD patients. This topic has been dealt with by Bilancio G and coworkers [8] who reviewed the few published studies which addressed this problem. The Authors report that a J-curve better describes the relationship between basal dietary protein intake and mortality, with about 33% excess mortality observed when protein intake was less than 0.8 g/kg of b.w. Based on these data, the Authors warn against the use of diets with protein restrictions below 0.8 g/kg.

Cozzolino M and coworkers [9] faced another very hot topic related to the emerging role of VitK deficiency as one among the leading causal factors for vascular calcification, which represent one of the most common findings in CKD patients, related to all-cause mortality and cardio-vascular related morbidity and mortality. The Authors reviewed the most updated aspects in this very critical problem, suggesting also which could be the potential corrective interventional approaches.

The high cardio-vascular risk of CKD patients recognizes a number of non-traditional risk factors, in addition to the traditional ones shared with the general population. Among the former group, hyperhomocysteinemia has been recognized as one of the possible causal factors, which could be corrected, at least in part, by folic acid supplementation. Irene Capelli et al. [10] carefully reviewed the most relevant studies dealing with this topic. Though the available literature does not give strong evidence that the correction of the increased homocysteine serum levels is associated with a clear improvement of the cardio-vascular outcomes in CKD patients, the Authors feel they can suggest that supplementation with folic acid could be of some utility, in particular in patients with more advanced CKD.

The importance of magnesium, both in physiology and in pathological conditions, has gained increasing interest in recent decades. Both hypo- and hypermagnesia can be associated with relevant cardio-vascular, neurological, skeletal, and other metabolic changes. CKD patients may present either increased or reduced serum levels of magnesium, depending on the stage of disease, dietary intake, the use of drugs affecting the metabolism of this cation or the dialysis treatment. This intriguing problem is nicely addressed in this Issue of *Nutrients* by Nicoline H. J. Leenders and Marc G. Vervloet [11], giving the readers a very complete overview on the topic.

In recent years, more and more emphasis has been given to the potential role of intestinal MB in a wide range of clinical conditions. CKD, in particular in the most advanced stages, has been reported to be associated with changes of intestinal MB which in turn could contribute to the inflammatory and malnutrition status that is a common clinical feature of CKD patients. On this background, interventions directed to correct the intestinal dysbiosis associated with CKD have been proposed, aimed to correct also some uremic symptoms. Anna Pisano and colleagues [12] performed a systematic review and a metanalysis of 17 eligible studies directed to explore the effects of biotic treatment on some clinical and biochemical endpoints in CKD patients. Due also to the large heterogeneity and the low quality of these studies, the Authors concluded that the available evidence does not support the indication to recommend biotic treatment in the clinical set of CKD.

The issue of gut MB in CKD has been also dealt with by Denise Mafra et al. [13], with a very extensive and thorough review of the literature. The Authors mainly focused on dietary patterns and some bioactive compounds that may modulate gut MB, generating hypotheses and suggesting the background and the rationale for future studies on the potential effects of nutritional interventions directed to correct the gut dysbiosis in CKD patients.

Patients with CKD are characterized by a high number of comorbidities. Therapeutic intervention in such patients is therefore addressed not only to the treatment of the underlying nephropathy, but also to the numerous metabolic and organic complications present.

Dietary intervention represents one of the most frequently used therapeutic possibilities in this clinical setting. In this field, however, there are still many obscure points regarding which elements to correct, whether in defect or excess, and what are the desired and undesired clinical effects of these corrections.

The articles published in this Issue of *Nutrients* offer the reader some interpretative hints and practical suggestions for intervention in a field that still remains largely to be explored.
